# P02.139. A comparative study of Chinese medicine and hormone therapy in the treatment of menopausal symptoms in perimenopausal and postmenopausal women

**DOI:** 10.1186/1472-6882-12-S1-P195

**Published:** 2012-06-12

**Authors:** H Azizi, Y Liu, L Du, C Wang, H Esmaily, H Bahrami-Taghanaki, H Azizi, M Soleimannejad, X Xue

**Affiliations:** 1 Mashhad University of Medical Sciences, Mashad, Iran; 2 Beijing University of Chinese Medicine, Beijing, China; 3 Peking University People Hospital, Beijing, China; 4 Islamic Azad University of Mashhad, Mashad, Iran; 5 Department of Cardiology, Imam Reza Hospital, Amol, Iran

## Purpose

To compare the therapeutic effect of a Chinese herbal medicine (CHM) named Kun Bao Wan, acupuncture and hormone therapy (HT) on menopause related symptoms of peri- and postmenopausal women.

## Methods

Fifty seven Chinese women completed 2 months of treatment with either CHM (5 gr BID, n=22), acupuncture in conjunction with CHM (CHM 5 gr BID + 10 sessions of acupuncture, n=20) or hormone therapy (n=15). Clinical symptoms were assessed by Kupperman index. Levels of FSH, estradiol, and symptom intensity and count were measured before and after the treatment.

## Results

CHM, acupuncture+CHM and hormone therapy significantly decreased Kupperman score (p<0.001 in each group) and symptom count (p<0.05). The mean difference in Kupperman score between baseline and 2 months among the 3 groups was significantly varied (p=0.02) with better results for acupuncture+CHM compared with CHM alone. Acupuncture+CHM as well as hormone therapy significantly reduced the level of FSH (p<0.05), but CHM alone didn’t cause any significant decrease in the level of FSH (p>0.05). The mean difference in the level of FSH between baseline and 2 months among the 3 groups was significantly different (p=0.02) with significantly better results for HT compared to CHM. The 3 treatments did not make any significant increase in the level of E2 (p>0.05). In postmenopausal women, the effect of HT and acupuncture+CHM were significantly better than CHM alone (p<0.05) whilst in perimenopausal women they were the same.

## Conclusion

The combination of Chinese herbal medicine and acupuncture proved as effective as hormone therapy in the treatment of menopausal symptoms, and it achieved better outcomes than herbal medicine alone, especially in postmenopausal women.

